# Rapidly Progressive Acute Limb Ischemia With Irreversible Gangrene and Major Amputation in a Resource-Limited Setting: A Case Report

**DOI:** 10.7759/cureus.116399

**Published:** 2026-09-17

**Authors:** Sai Say Han

**Affiliations:** 1 Department of Medicine, East Yangon General Hospital, Yangon, MMR; 2 Department of Medicine, University of Medicine 1, Yangon, MMR

**Keywords:** acute limb ischemia, acute lower limb ischemia, arterial embolism, atrial fibrillation, foot gangrene, irreversible ischemia, major limb amputation, peripheral arterial diseases, reperfusion injury

## Abstract

Acute limb ischemia (ALI) is a vascular emergency in which delayed recognition and treatment can result in irreversible tissue necrosis, gangrene, limb loss, and potentially life-threatening systemic complications. We present the case of a 63-year-old woman with atrial fibrillation and heart failure who developed acute lower-limb ischemia with progressive discoloration and tissue necrosis. Lower-extremity arterial Doppler ultrasonography demonstrated extensive arterial occlusive disease with absent flow across multiple major arterial segments, confirming severe limb ischemia. Serial clinical photographs documented progressive cyanotic and violaceous discoloration with later bullous formation and worsening ischemic tissue injury in the setting of established gangrene. Vascular assessment concluded that the affected limb was no longer salvageable by revascularization. Because reperfusion of extensively necrotic tissue was considered to carry substantial risk of local and systemic reperfusion complications, major limb amputation was recommended. The patient subsequently underwent definitive amputation of the nonviable limb. This case illustrates the narrow therapeutic window in ALI and emphasizes the importance of early recognition, prompt vascular assessment, and timely revascularization before irreversible ischemia develops. Once extensive tissue necrosis and established gangrene occur, primary amputation may represent the appropriate definitive management when limb salvage is no longer feasible.

## Introduction

Acute limb ischemia (ALI) is a vascular emergency caused by a sudden reduction in arterial perfusion that threatens limb viability and can rapidly progress to irreversible tissue injury, gangrene, major amputation, and death [1,2]. Clinical manifestations classically include pain, pallor, pulselessness, paresthesia, paralysis, and poikilothermia, although not all findings are present in every patient [3,4]. Early recognition and urgent vascular assessment are therefore critical because the likelihood of successful limb salvage decreases as the duration and severity of ischemia increase [1-4].

The principal mechanisms of ALI include in situ arterial thrombosis and arterial embolism. Cardiac embolization is an important cause, particularly in patients with atrial fibrillation, intracardiac thrombus, valvular heart disease, or impaired ventricular function [2,3]. Atrial fibrillation is particularly relevant because of its established association with systemic thromboembolic events, including peripheral arterial embolization [2,5]. Duplex ultrasonography and computed tomography angiography can help define the location and extent of arterial obstruction, while the clinical assessment of limb viability remains central to determining the urgency and type of intervention [2,3].

Revascularization by surgical or endovascular techniques can preserve a threatened but viable limb when performed promptly. However, once ischemia has progressed to irreversible tissue necrosis and established gangrene, restoration of blood flow may no longer provide meaningful limb salvage and can expose the patient to complications associated with reperfusion of extensively ischemic tissue. In such circumstances, major amputation may become the appropriate definitive treatment [1,2]. Although Doppler ultrasonography demonstrated extensive multilevel arterial occlusive disease, the available data could not definitively distinguish an acute embolic event from acute thrombosis superimposed on pre-existing peripheral arterial disease. Serial assessment of limb viability therefore remained central to determining whether meaningful revascularization was still possible. We report a case of severe acute lower-limb ischemia in a woman with atrial fibrillation and heart failure, with Doppler-confirmed extensive arterial occlusion and progression to established gangrene, in whom the limb was considered unsalvageable and major amputation was ultimately required. The case underscores the narrow therapeutic window in ALI and the consequences of progression to irreversible ischemia.

## Case presentation

A 63-year-old woman with a medical history significant for atrial fibrillation and heart failure presented with acute ischemic changes involving the right lower extremity. The affected limb developed progressive pain, coolness, cyanotic discoloration, and distal tissue compromise. On examination, there was marked asymmetry compared with the contralateral limb, with extensive dusky discoloration involving the distal right leg and foot, consistent with severe arterial insufficiency.

Electrocardiography demonstrated atrial fibrillation. Given the acute vascular presentation, arterial and venous Doppler ultrasonography of the lower extremities was performed. The common femoral arteries remained patent, although reduced-velocity biphasic waveforms were documented. More distally, absent or markedly impaired flow was identified in major arterial segments, including the superficial femoral, popliteal, anterior tibial, and posterior tibial arteries, consistent with extensive arterial occlusive disease and severe limb ischemia. Collateral vessels were also noted. The venous Doppler examination additionally demonstrated findings concerning for concomitant deep venous thrombosis.

Supportive medical management was initiated, including furosemide for concomitant heart failure. Based on the treating team’s recollection, subcutaneous enoxaparin 40 mg was administered in the setting of impaired renal function; however, the complete contemporaneous medication administration record was not available for retrospective verification.

Cardiovascular surgical consultation was obtained remotely on February 26 at approximately 4:00 PM after review of the available vascular assessment. The consulting surgeon documented established gangrene and considered the right lower extremity beyond meaningful salvage by revascularization, with concern that attempted reperfusion could result in local and systemic complications and worsen the patient’s heart failure and impaired renal function. Amputation was therefore recommended. Because a complete contemporaneous sensory and motor examination was not preserved, a Rutherford category could not be reliably assigned at the earliest presentation; by the time of cardiovascular surgical assessment, the findings were consistent with irreversible ischemia (Rutherford category III).

The ischemic process subsequently worsened, with increasing cyanotic and violaceous discoloration and progressive tissue injury of the right lower extremity. Serial clinical photographs documented increasing extent and severity of the ischemic and gangrenous changes, including distal tissue necrosis and the later development of multiple tense bullae over the lower leg, ankle, and foot (Figure 1).

**Figure 1:**
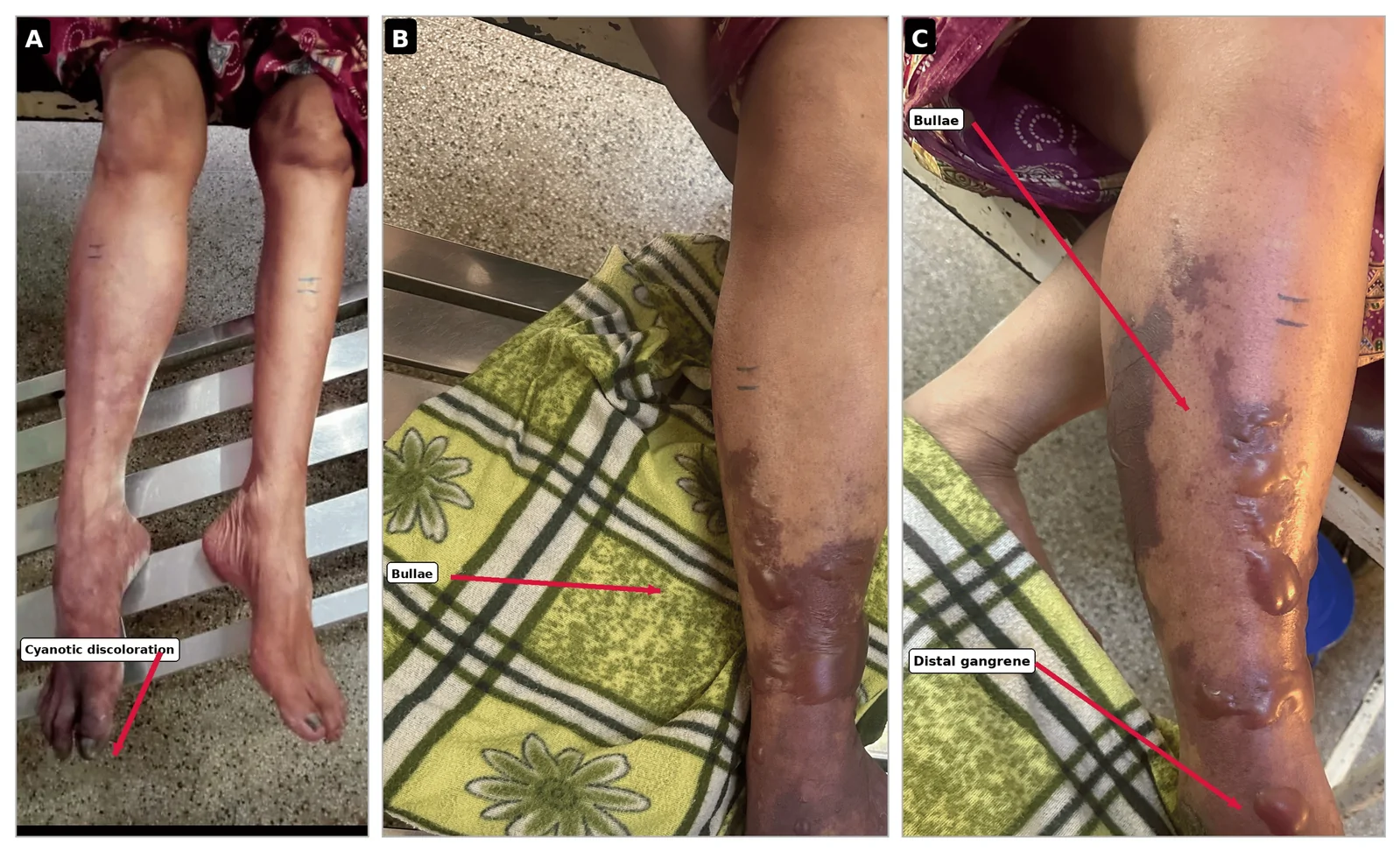
Serial progression of acute ischemic injury of the right lower extremity. (A) Distal cyanotic discoloration of the right lower extremity photographed on February 26 at 4:54 PM. (B) Markedly more extensive violaceous discoloration documented on March 6 at 12:09 PM. (C) Bullous formation with advanced ischemic tissue injury and distal gangrenous change documented on March 7 at 9:52 AM. Substantial documented deterioration occurred over approximately 22 hours between panels B and C. Times refer to photograph-capture times.

Vascular surgical assessment determined that the right lower extremity had progressed to irreversible ischemia with established gangrene and was no longer amenable to meaningful limb salvage by revascularization. Restoration of blood flow to the extensively ischemic and necrotic extremity was considered potentially hazardous because of the risk of severe local and systemic reperfusion complications. Major amputation was therefore recommended as definitive management.

The patient subsequently underwent a right above-knee amputation to remove the nonviable ischemic limb and prevent further systemic complications associated with progressive gangrene. Her postoperative course was clinically stable, and she was ultimately discharged alive following recovery from the procedure. Electrocardiography demonstrated an irregularly irregular rhythm compatible with atrial fibrillation (Figure 2).

**Figure 2:**
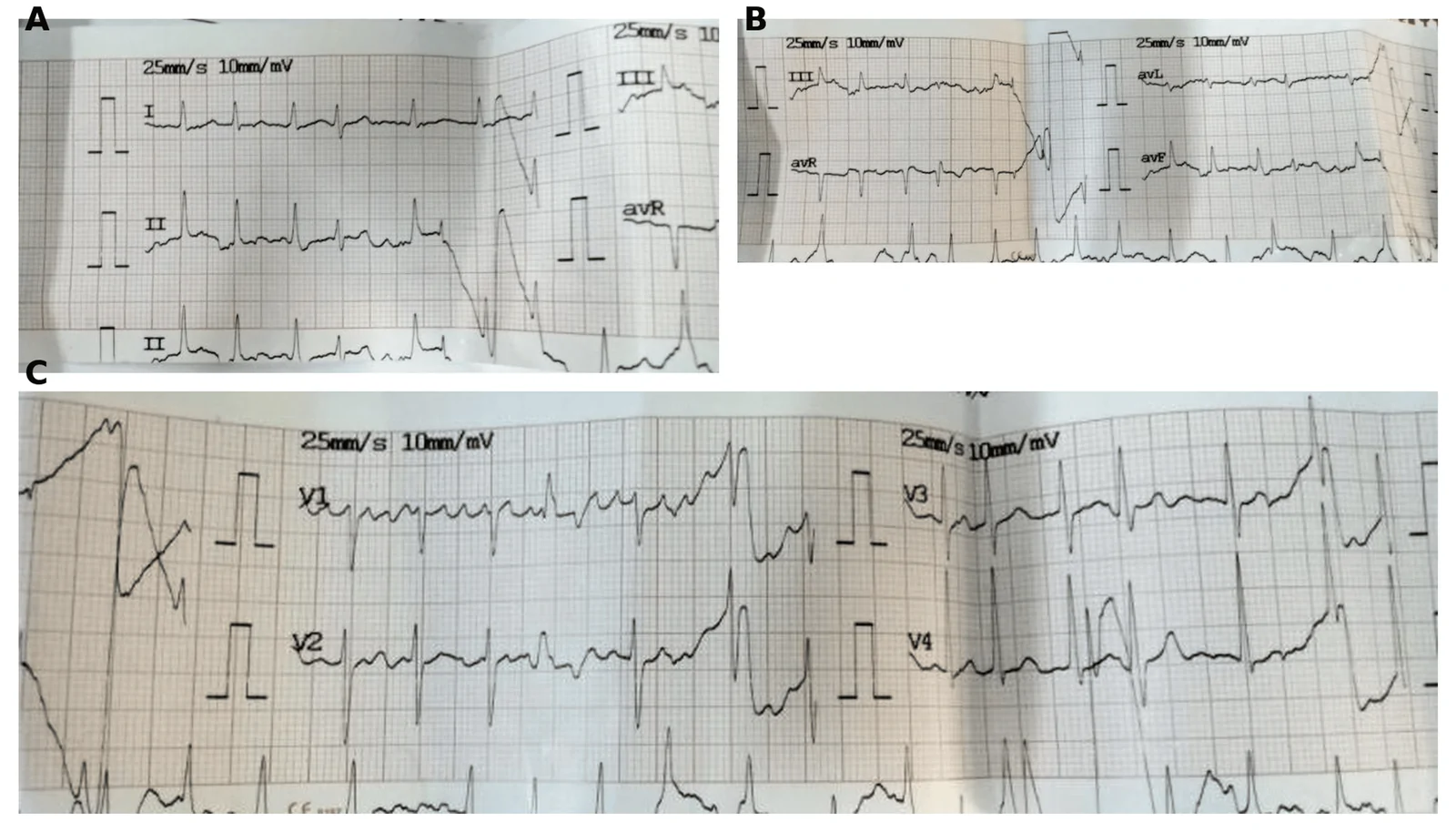
Electrocardiogram demonstrating atrial fibrillation. (A-C) Representative electrocardiographic tracings demonstrating an irregularly irregular ventricular rhythm without consistently identifiable P waves, compatible with atrial fibrillation.

Lower-extremity arterial and venous Doppler ultrasonography demonstrated extensive multilevel arterial occlusive disease, with preserved but abnormal flow in the common femoral arteries and absent flow in multiple distal arterial segments. Collateral arterial circulation was present, and the venous examination additionally documented deep venous thrombosis (Table 1).

**Table 1:** Lower-extremity arterial and venous Doppler ultrasonography findings. Lower-extremity arterial and venous Doppler ultrasonography demonstrated extensive multilevel arterial occlusive disease with absent or markedly reduced flow in distal arterial segments, together with collateral circulation and concomitant deep venous thrombosis. The right lower extremity showed the clinically severe ischemic progression leading to gangrene and subsequent above-knee amputation. DVT = deep venous thrombosis; USG = ultrasonography

Vascular structure	Doppler finding
Right common femoral artery	Patent; low-velocity biphasic waveform
Left common femoral artery	Patent; low-velocity biphasic waveform
Superficial femoral arteries	No patency demonstrated; absent color flow/significant waveform
Popliteal arteries	No patency demonstrated; absent color flow/significant waveform
Anterior tibial arteries	No patency demonstrated; absent color flow/significant waveform
Posterior tibial arteries	No patency demonstrated; absent color flow/significant waveform
Collateral arterial circulation	Collateral arteries visualized
Venous system	Abnormal venous findings with noncompressibility and absent color flow in affected segments
Overall Doppler impression	Extensive peripheral arterial occlusive/ischemic disease with concomitant deep venous thrombosis

## Discussion

ALI is a time-sensitive vascular emergency caused by an abrupt reduction in arterial perfusion that places limb viability at immediate risk. The clinical course may progress from pain, pallor, pulselessness, coolness, and sensory disturbance to motor dysfunction, tissue necrosis, and gangrene. The 2024 American College of Cardiology/American Heart Association guideline classifies ALI according to limb viability: a viable limb is classified as Rutherford category I, a threatened but salvageable limb as category II, and an irreversibly damaged, nonsalvageable limb as category III [1]. Prompt assessment is therefore critical because the opportunity for limb salvage decreases as the duration and severity of ischemia increase.

ALI may result from arterial embolism, in situ thrombosis, thrombosis of a previously treated arterial segment, trauma, aneurysmal disease, or other systemic thrombotic conditions. Cardiac disease remains an important potential source of arterial embolization. In particular, atrial fibrillation is strongly associated with systemic thromboembolic events and should raise suspicion for an embolic mechanism in a patient presenting with sudden limb ischemia [1,2,5]. In the present case, electrocardiography demonstrated atrial fibrillation, while Doppler ultrasonography confirmed extensive arterial occlusive disease involving multiple lower-extremity arterial segments. Duplex ultrasonography is useful in ALI because it can identify the location and extent of arterial obstruction, assess flow proximal and distal to the occlusion, and assist in planning further management [2].

Although atrial fibrillation provided a plausible cardioembolic substrate in this patient, the precise mechanism of the arterial occlusion cannot be established with certainty. No computed tomography angiography, catheter angiography, retrieved embolic material, or documented intracardiac thrombus was available to definitively establish a cardiac embolic source. Furthermore, the Doppler findings suggested extensive multilevel arterial disease rather than an isolated focal occlusion. Therefore, an embolic event, acute thrombosis superimposed on pre-existing peripheral arterial disease, or a combination of mechanisms should be considered. This distinction is important because ALI caused by embolism and ALI resulting from thrombosis of an atherosclerotic artery can present similarly but may differ in vascular anatomy, collateral circulation, and therapeutic approach [2].

A particularly important aspect of this case was the rapid progression of ischemia in a hospital with limited access to definitive vascular intervention. The treating hospital had general surgical services but lacked an on-site cardiovascular/vascular surgical or endovascular revascularization capability. The patient also required medical stabilization for concomitant heart failure, atrial fibrillation, and impaired renal function. A formal transfer to a tertiary vascular center was not undertaken before remote cardiovascular surgical consultation. By the time cardiovascular surgical assessment was obtained, the limb had already progressed to established gangrene and was considered beyond meaningful salvage by revascularization. The absence of immediate on-site revascularization capability therefore constrained the available limb-salvage options, although the retrospective records do not permit attribution of progression to resource limitation alone.

The patient’s ischemic changes progressed rapidly, with worsening cyanotic and violaceous discoloration, increasing distal tissue necrosis, and later bullous formation in an extremity already assessed as having established gangrene. The serial clinical photographs in this case demonstrate this evolution clearly. These findings, together with the subsequent vascular assessment that the extremity was no longer salvageable, were compatible with advanced irreversible ischemic injury. The case illustrates an important practical characteristic of ALI: the therapeutic window for revascularization can be narrow, particularly in severe occlusion with insufficient collateral perfusion. Reviews of ALI management similarly emphasize that the longer severe ischemia persists, the lower the likelihood that functional limb salvage can be achieved [1,2].

Resource availability becomes particularly relevant in such circumstances. Revascularization strategies for salvageable ALI include surgical thromboembolectomy, catheter-directed thrombolysis, mechanical thrombectomy, bypass surgery, and combinations of open and endovascular techniques. The most appropriate strategy depends not only on the etiology, anatomical location, and severity of ischemia but also on the expertise and facilities available at the treating institution [1,2]. In centers without immediate vascular surgical or endovascular capability, early recognition and rapid referral or transfer become essential components of treatment. The present case demonstrates how limited access to specialized vascular intervention, occurring in the setting of rapidly progressive ischemia, can substantially constrain opportunities for limb salvage.

However, once extensive tissue necrosis and established gangrene are present, the balance between attempted revascularization and definitive amputation changes. An irreversibly ischemic extremity is considered nonsalvageable, and restoration of arterial flow may no longer restore useful tissue viability. Reperfusion of profoundly ischemic or necrotic tissue may additionally release potassium, hydrogen ions, myoglobin, and other ischemic metabolites into the systemic circulation, potentially contributing to metabolic derangement, acute kidney injury, cardiovascular instability, compartment syndrome, and multiorgan dysfunction [1,2]. Thus, attempts at revascularization after irreversible injury may expose the patient to systemic reperfusion complications without providing meaningful limb salvage.

In this patient, vascular assessment concluded that the right lower extremity had progressed beyond the point at which meaningful revascularization could be achieved. The presence of extensive ischemic tissue injury and established gangrene supported definitive major amputation rather than a late attempt at restoring arterial perfusion. A right above-knee amputation was subsequently performed to remove the nonviable extremity and mitigate the risk of further local and systemic complications. The patient remained clinically stable following surgery and was discharged alive. Although major amputation carries substantial functional and psychosocial consequences, it may become the appropriate definitive intervention when irreversible ischemia has already occurred and continued attempts at limb salvage would provide little clinical benefit.

Another notable feature of this case was the reported coexistence of arterial occlusive disease and deep venous thrombosis. Venous thrombosis is not ordinarily the primary mechanism of classical ALI, and its presence may reflect a broader thrombotic milieu or accompanying vascular pathology. Severe venous obstruction can itself compromise limb circulation in extreme circumstances, although the patient’s dominant clinical presentation and arterial Doppler abnormalities were consistent with severe arterial ischemia [2]. The coexistence of atrial fibrillation, extensive arterial abnormalities, and venous thrombosis therefore highlights the complexity of assigning a single thrombotic mechanism in the absence of advanced vascular imaging or definitive embolic-source documentation.

This case has several limitations. Advanced arterial imaging with computed tomography angiography or catheter angiography was not available, and therefore the precise anatomy and etiology of the occlusion could not be fully characterized. Echocardiographic evidence of an intracardiac thrombus was also not available to establish atrial fibrillation as the definitive source of arterial embolization. In addition, access to immediate vascular revascularization was limited by the resources of the treating hospital. Nevertheless, the combination of objective Doppler evidence, serial clinical photographs, vascular surgical assessment, and the ultimate need for major amputation provides a clear demonstration of rapidly progressive severe limb ischemia.

The principal lesson from this case is that timely assessment and access to definitive vascular care are important considerations in the management and outcome of ALI. Early recognition, prompt assessment of limb viability, immediate anticoagulation when appropriate, early vascular consultation, and timely surgical or endovascular revascularization offer the greatest opportunity for preserving a threatened limb. When such resources are unavailable locally, rapid communication and transfer pathways to a vascular-capable center should be considered as early as possible. Conversely, once the limb has progressed to irreversible ischemia with established gangrene, the objective of treatment must shift from limb salvage to definitive management of the nonviable extremity and prevention of further systemic complications.

## Conclusions

ALI is a time-critical vascular emergency in which progression to irreversible tissue injury may result in gangrene and major limb loss. This case demonstrates rapidly progressive severe right lower-extremity ischemia in a patient with atrial fibrillation and extensive arterial occlusive disease. The precise mechanism of arterial occlusion could not be definitively established. The treating hospital did not have immediate on-site vascular revascularization capability; however, the retrospective records do not permit determination of the extent to which this limitation contributed to the eventual limb loss. By the time cardiovascular surgical assessment was obtained, established gangrene was documented and the limb was considered nonsalvageable by revascularization, making right above-knee amputation the definitive treatment. Early recognition of ALI, prompt assessment of limb viability, timely vascular consultation, and access to definitive vascular care remain essential. Once irreversible ischemia has developed, treatment priorities must shift from limb salvage to definitive management of the nonviable extremity and prevention of systemic complications.

## References

[1] Gornik HL, Aronow HD, Goodney PP (2024). 2024 ACC/AHA/AACVPR/APMA/ABC/SCAI/SVM/SVN/SVS/SIR/VESS Guideline for the Management of Lower Extremity Peripheral Artery Disease: a report of the American College of Cardiology/American Heart Association Joint Committee on Clinical Practice Guidelines. Circulation.

[2] Olinic DM, Stanek A, Tătaru DA, Homorodean C, Olinic M (2019). Acute limb ischemia: an update on diagnosis and management. J Clin Med.

[3] Björck M, Earnshaw JJ, Acosta S (2020). Editor's choice - European Society for Vascular Surgery (ESVS) 2020 Clinical Practice Guidelines on the Management of Acute Limb Ischaemia. Eur J Vasc Endovasc Surg.

[4] Rosenberg H, Rosenberg E, Kubelik D (2023). Acute limb ischemia. CMAJ.

[5] Van Gelder IC, Rienstra M, Bunting KV (2024). 2024 ESC Guidelines for the management of atrial fibrillation developed in collaboration with the European Association for Cardio-Thoracic Surgery (EACTS). Eur Heart J.

